# Supplementary material for: Sustainable Engineered Design and Scalable Manufacturing of Upcycled Graphene Reinforced Polylactic Acid/Polyurethane Blend Composites Having Shape Memory Behavior
Source: Polymers (Basel). 2023 Feb 21;15(5):1085. doi: 10.3390/polym15051085 (PMC10007146; doi:10.3390/polym15051085)
Supplement: Supplementary file 1 [file polymers-15-01085-s001.zip › polymers-2187316-supplementary.pdf]

polymers

Supplementary Materials

Sustainable Engineered Design and Scalable Manufacturing of Upcycled Graphene Reinforced Polylactic Acid/Polyurethane Blend Composites Having Shape Memory Behavior

Busra Cetiner [1,2], Gulayse Sahin Dundar [1,2], Yusuf Yusufoglu [3] and Burcu Saner Okan [1,2,*]

1 Sabanci University Integrated Manufacturing Technologies Research and Application Center & Composite Technologies Center of Excellence, Teknopark Istanbul, Istanbul 34906, Turkey
2 Faculty of Engineering and Natural Sciences, Materials Science and Nanoengineering, Sabanci University, Istanbul 34956, Turkey
3 Adel Kalemcilik Ticaret ve Sanayi A.S.,, Kocaeli 41480, Turkey
* Correspondence: author: bsanerokan@sabanciuniv.edu

In the present study, thermoplastic polyurethane and polylactic acid were melt mixed via thermokinetic mixer. Figure S1 represents FT-IR spectra of neat TPU and PLA and their optimum blendcomposition. PLA has C=O stretching bend at 1746 cm^{-1}, C–H bending at 1454 cm^{-1} and 1358 cm^{-1} and C–O stretching vibrations at 1181 cm^{-1}, 1098 cm^{-1}, 1082 cm^{-1} and 1043 cm^{-1} [58]. TPU has shown NH stretching vibration at 3332 cm^{-1}, aliphatic asymmetric and symmetric CH_2 stretching vibrations at 2955 cm^{-1}, 2918 cm^{-1}, 2873 cm^{-1} and 2850 cm^{-1}, C=O stretching vibration of ester groups at 1726 cm^{-1}, 1701 cm^{-1} and 1637 cm^{-1}, C–N stretching and bending of urethane group at 1596 cm^{-1}, 1528 cm^{-1} and 1462 cm^{-1}, CH_2 deformation vibration 1413 cm^{-1}, 1393 cm^{-1} and 1358 cm^{-1}, C–N stretching vibration at 1309 cm^{-1}, C–O–C stretching of ester bonds at 1252 cm^{-1} and 1219 cm^{-1}, C=C stretching at 1167 cm^{-1}, 1139 cm^{-1}, 1075 cm^{-1} and 1018 cm^{-1} [59,60]. FT-IR spectrum of PLA:TPU = 9:1 blend composite has the characteristics peaks of PLA and TPU polymers.

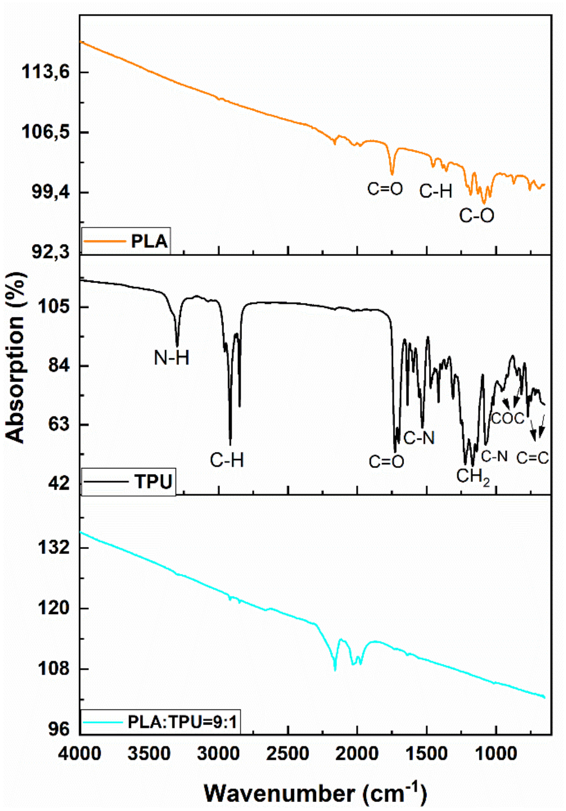

Figure S1. FT-IR spectra of PLA, TPU and PLA:TPU = 9:1 blend composite.

In present study, waste-tire derived graphene nanoplatelets (GNP) produced through upcycling and recycling processes have utilized as a reinforcing agent of TPU and PLA polymer matrices. Table S1 summarizes chemical composition of GNP obtained from X-Ray Photoelectron Spectroscopy (XPS) characterization.

Table S1. XPS survey scan spectrum of GNP.

	C (at%)	O (at%)	Other (at%)
GNP	87.4	9.1	3.5

Figure S2 demonstrates the TEM image and Raman spectrum of GNP. Specific peaks including the D and G peaks of GNP were shown at 1350 cm^{-1} and 1580 cm^{-1}, respectively. TEM image of GNP has demonstrated that the average length of platelet of GNP has evaluated 50 ± 4 nm.

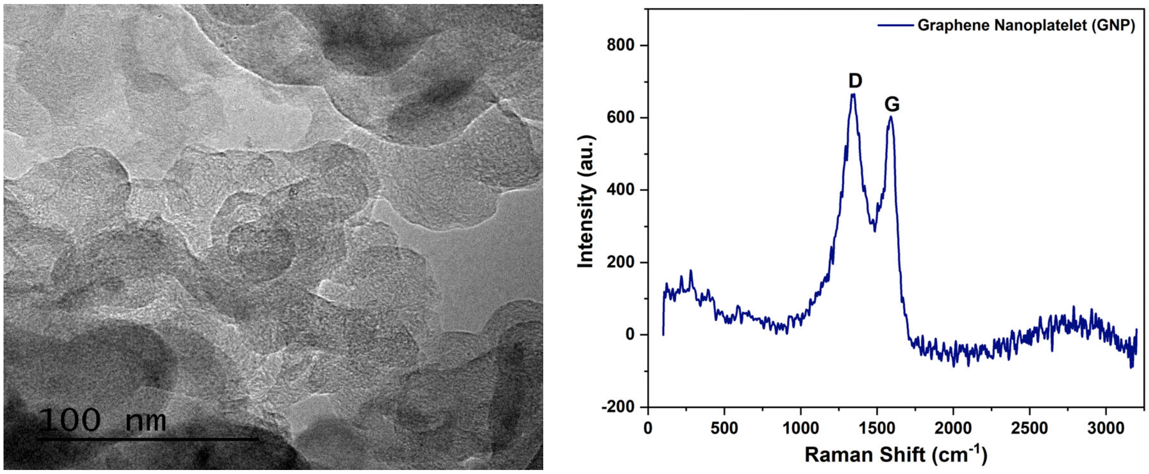

(**a**) (**b**)

Figure S2. (**a**) TEM image and (**b**) Raman spectrum of waste-tire derived GNP.

Figure S3 displays X-Ray Diffraction (XRD) pattern of GNP. Waste-tire derived GNP shows unique (002) plane and implies 21.5% of crystallinity [61].

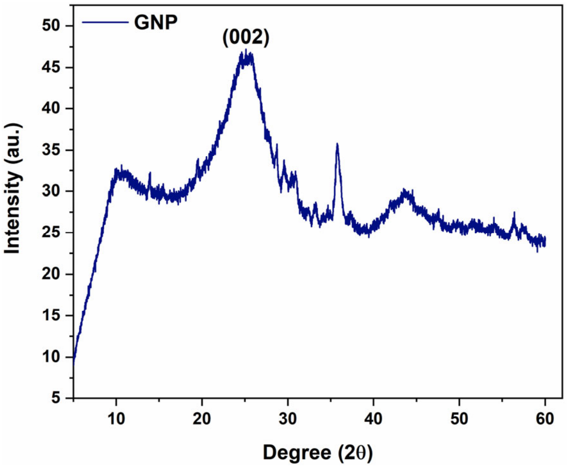

Figure S3. XRD pattern of GNP.

The crystallization percentage of composites is calculated by using the following crystallization equation as below:

$$Xc = \left(\frac{\Delta H_M}{\Delta H_M^{100\%}}\right) \times 100 \quad \text{(S1)}$$

where ΔH_M is the melting enthalpy of the present TPU sample and $\Delta H_M^{100\%}$ is the melting enthalpy of 100% crystalline TPU. The melting enthalpy of 100% crystalline TPU is calculated as 8.49 J/g [20].

Figure S4 shows the secondary heating and cooling curves of GNP based PLA composites. It is stated that there is no sharp change in the secondary heating behavior of GNP based PLA composites when compared to neat PLA. On the other hand, the crystallization degree has changed by the addition of GNP in PLA matrix, and this confirmed that GNP

acts as a nucleating agent in PLA but does not have a drastic effect on mechanical properties of PLA. GNP preserves the mechanical properties of PLA but enhance the crystallinity degree which is important for the injection molding of the produced composites.

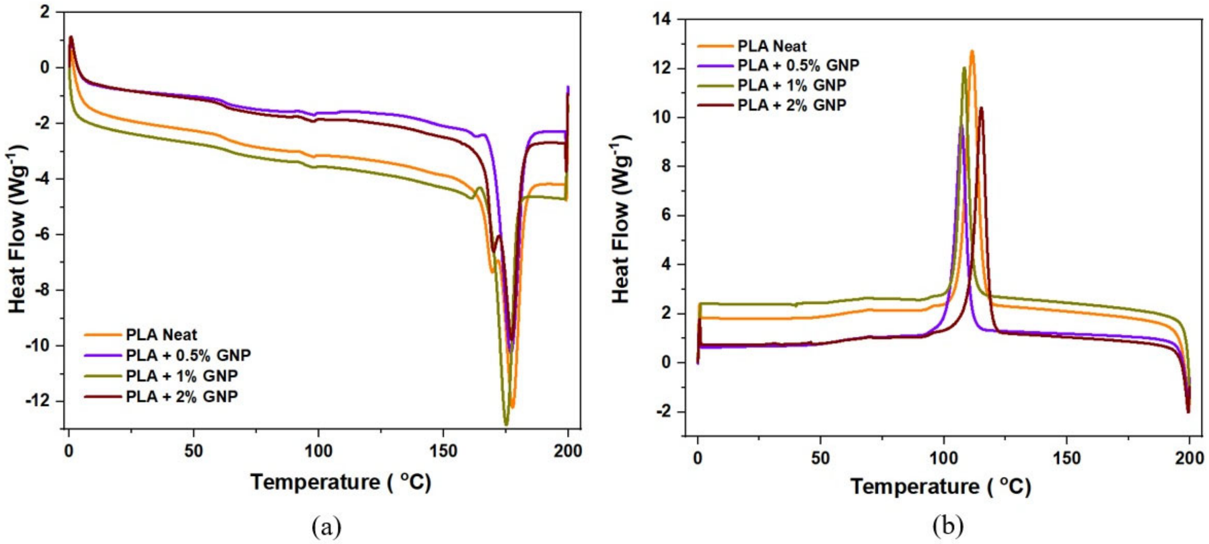

Figure S4. (**a**) Secondary heating curves and (**b**) cooling curves of GNP reinforced PLA composites.

Table S2 shows the thermal properties of PLA-based GNP loaded composites from DSC results. 6 °C in T_g, 3 °C in T_m and 12 °C of T_c improvements as well as enhancements in enthalpies were achieved. The enthalpies of the melting and crystallization increased by the addition of GNP due to the nucleation effect of GNP and risen the thermal properties.

Table S2. Summary of thermal properties of GNP reinforced PLA composites.

Sample	Glass Transition Temperature T_g (°C)	Melting Temperature T_m (°C)	Melting Enthalpy ΔH_M (J/g)	Crystallization Temperature T_c (°C)	Crystallization Enthalpy ΔH_c (J/g)
Neat PLA	62.27	177.65	−39.95	106.88	32.32
PLA + 0.5% GNP	66.30	176.86	−41.58	110.70	32.93
PLA + 1% GNP	68.30	177.09	−42.77	111.65	41.71
PLA + 2% GNP	66.95	180.04	−44.95	118.67	34.11

To calculate the crystallization percentage of PLA based GNP reinforced composites, 100% crystalline PLA should be divided into obtained melting enthalpies, as seen in the TPU composite section. The melting enthalpy of 100 crystalline PLA was calculated 93.7 J/g [62]. Table S3 shows the crystallinity degree of PLA-based GNP loaded composites, indicating that GNP loading increased the total crystallinity degree.

Table S3. Crystallinity and amorphous percentages of PLA based composites as a function of GNP content with melting enthalpy values.

Sample	Melting Enthalpy ΔH_M (J/g)	ΔH_M^{100} (J/g)	Crystallinity (%)	Amorphous (%)
Neat PLA	−39.95	93.7	43	57
PLA+0.5%GNP	−41.58	93.7	44	56
PLA+1%GNP	−42.77	93.7	45	55
PLA+2%GNP	−44.95	93.7	48	52

Table S4 summarizes the tensile properties of PLA:TPU = 9:1 based GNP integrated composites. The tensile properties of blend composite by adding GNP showed that a drop in the tensile strength and modulus due to laminar structure of PLA:TPU = 9:1 blend. Considering the results of the tensile properties, 0.5% GNP addition to the blend had the optimum results.

Table S4. Summary of tensile properties of PLA:TPU = 9:1 based composites with different GNP loading ratios.

Sample	Tensile Strength (MPa)	Improvement of Tensile Strength (%)	Tensile Modulus (MPa)	Improvement of Tensile Modulus (%)	Elongation at break (%)
PLA:TPU = 9:1	52.35 ± 1.23	-	3031 ± 99.61	-	2.80 ± 0.29
PLA:TPU = 9:1+ 0.5% GNP	51.55 ± 2.97	−2	2793 ± 62.67	−8	3.40 ± 1.64
PLA:TPU = 9:1 +1% GNP	49.60 ± 3.97	−5	2858 ± 115.33	−6	3.03 ± 1.57
PLA:TPU = 9:1 +2% GNP	48.98 ± 4.08	−6	2775 ± 183.87	−8	3.98 ± 1.67
